# Unravelling the Allosteric Targeting of PHGDH at the ACT-Binding Domain with a Photoactivatable Diazirine Probe and Mass Spectrometry Experiments †

**DOI:** 10.3390/molecules26020477

**Published:** 2021-01-18

**Authors:** Quentin Spillier, Séverine Ravez, Simon Dochain, Didier Vertommen, Léopold Thabault, Olivier Feron, Raphaël Frédérick

**Affiliations:** 1 Medicinal Chemistry Research Group (CMFA), Louvain Drug Research Institute (LDRI), Université Catholique de Louvain, B-1200 Brussels, Belgium; Quentin.Spillier@nyulangone.org (Q.S.); simondochain@hotmail.com (S.D.); leopold.thabault@uclouvain.be (L.T.); 2 Pole of Pharmacology and Therapeutics (FATH), Institut de Recherche Expérimentale et Clinique (IREC), Université Catholique de Louvain, B-1200 Brussels, Belgium; olivier.feron@uclouvain.be; 3 UMR-S1172-JPArc-Centre de Recherche Jean-Pierre AUBERT Neurosciences et Cancer, Inserm, CHU Lille, Université de Lille, F-59000 Lille, France; severine.ravez@univ-lille.fr; 4 de Duve Institute, Université Catholique de Louvain, B-1200 Brussels, Belgium; didier.vertommen@uclouvain.be

**Keywords:** PHGDH, diazirine, photoaffinity labeling

## Abstract

The serine biosynthetic pathway is a key element contributing to tumor proliferation. In recent years, targeting of phosphoglycerate dehydrogenase (PHGDH), the first enzyme of this pathway, intensified and revealed to be a promising strategy to develop new anticancer drugs. Among attractive PHGDH inhibitors are the α-ketothioamides. In previous work, we have demonstrated their efficacy in the inhibition of PHGDH in vitro and in cellulo. However, the precise site of action of this series, which would help the rational design of new inhibitors, remained undefined. In the present study, the detailed mechanism-of-action of a representative α-ketothioamide inhibitor is reported using several complementary experimental techniques. Strikingly, our work led to the identification of an allosteric site on PHGDH that can be targeted for drug development. Using mass spectrometry experiments and an original α-ketothioamide diazirine-based photoaffinity probe, we identified the 523Q-533F sequence on the ACT regulatory domain of PHGDH as the binding site of α-ketothioamides. Mutagenesis experiments further documented the specificity of our compound at this allosteric site. Our results thus pave the way for the development of new anticancer drugs using a completely novel mechanism-of-action.

## 1. Introduction

Many recent findings highlighted the importance of serine metabolism in cancer [1,2,3,4]. Given that serine is a key metabolite to support cell proliferation, an increase in serine supply is required to sustain cancer progression. Serine can be taken up from the extracellular environment or produced by the de novo serine synthesis pathway (SSP) starting from the glycolytic metabolite 3-phosphoglycerate (3-PG). The SSP is composed of three enzymes: phosphoglycerate dehydrogenase (PHGDH) that converts 3-PG into 3-phosphohydroxypyruvate, phosphoserine-amino transferase (PSAT-1) converting 3-phosphohydroxypyruvate into phosphoserine, and phosphoserine phosphatase (PSPH) eventually catalyzing the dephosphorylation of phosphoserine into serine.

**Figure 1:**
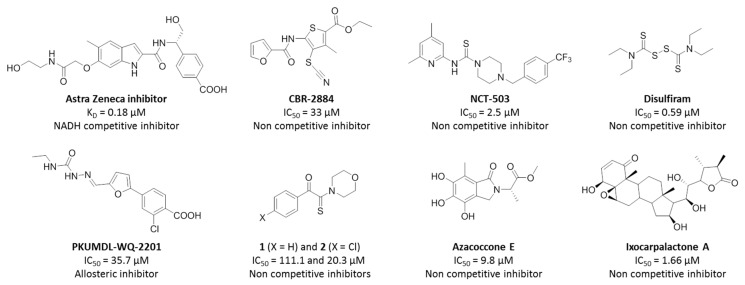
Overview of some reported PHGDH inhibitors.

In 2011, two independent publications highlighted the oncogenic role of PHGDH [5,6]. Since then, seminal publications confirmed the importance of PHGDH in cancer (triple negative ER breast cancer, glioma, pancreatic cancer, etc.) [7,8,9] and notably demonstrated that PHGDH extinction led to a significant reduction in tumor proliferation [10]. Given the potential of PHGDH as an attractive anticancer drug target, research efforts were devoted to identify potent PHGDH inhibitors (Figure 1) [10,11,12,13,14,15,16,17]. As depicted in Figure 1, besides indole derivative developed by Astra Zeneca as NADH competitive inhibitors, all reported molecules were shown to act as non-competitive inhibitors and are characterized with PHGDH inhibitory potency in the micromolar range. Indeed, the high physiological concentration of NADH (0.3 mM) hampers the design of competitive inhibitors. On the other hand, the development of non-competitive, allosteric PHGDH inhibitors is a promising approach, notably to overcome the problem of specificity against other NAD-dependent enzymes.

Until recently, only two different allosteric sites were identified for PHGDH, the ASB (allosteric substrate binding) and the ACT (aspartate-kinase chorismate-mutase-tyrA) domains. These two domains, located at the C-terminal part of the protein, have, up to date, never been intentionally targeted to develop PHGDH inhibitors, and their role in the control of PHGDH activity remains poorly understood. In 2016, Wang and coworkers suggested two other allosteric sites of PHGDH that were confirmed by the use of probes targeting these sites. The first, sharing at least five amino acids with the enzyme active site (Gly 78, Val 79, Asp 80, Asn 81, and Val 82), is located at the interface of the enzyme active site and NAD binding domain, whereas the second, smaller, was identified in the substrate-binding cavity [13]. More recently, Zheng and coworkers suggested another potential allosteric site located at the back-side of the active site and which could be the site of action of the PHGDH inhibitor Ixocarpalactone A [17]. Finally, we have recently reported an inhibition mechanism of PHGDH which involves disrupting its active oligomerization state using disulfiram (DSF), a well-known anti-alcohol agent. DSF inhibits PHGDH through oxidation of a specific cysteine (Cys116) located at the interface between two PHGDH monomers [15].

**Figure 2:**
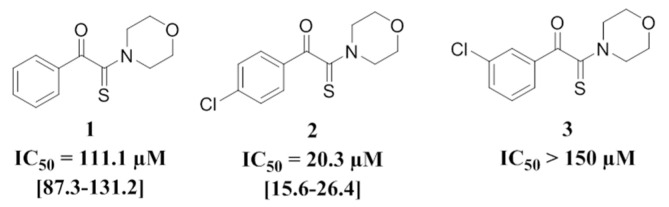
α-ketothioamide inhibitors and preliminary SAR investigations.

These examples demonstrate the importance of detailing the mechanism-of-action of newly developed PHGDH inhibitors to better understand the mechanisms involved in PHGDH regulation and nurture the development of new inhibitors. We recently reported a convergent pharmacophore strategy that led to the identification of the α-ketothioamide **1** (Figure 2) endowed with a PHGDH inhibitory potency in the 100 µM range [14]. A preliminary round of optimization around this hit led to the design of **2** exhibiting a five-fold improved IC_50_ value of 20.3 µM.

In the present work, the site-of-action of compound **2** was investigated to identify novel PHGDH allosteric site and possibly demonstrate that this site can be targeted to design new anticancer therapies.

## 2. Results and Discussion

### 2.1. Biophysical Characterization of the Lead Compound

Previous studies in our laboratory highlighted compound **1** and the *para*-substituted analogue **2** as promising PHGDH inhibitors. Early investigations of the structure-activity relationships (SARs) revealed the importance of this *para*-substitution pattern (Figure 2, compare the *para*-chlorinated derivative **2** and the *meta*-chlorinated analogue **3**, for instance) [14]. Moreover, recent studies also demonstrated that structural modifications of the linker and/or the morpholino ring are poorly tolerated and only lead to improved inhibitory potency when coupled with chlorinated *para*-substitution [18]. However, at this stage, the precise binding mode of **2** to PHGDH remained mostly unclear. We previously reported that this series act with a non-competitive mode of inhibition with respect to both substrates and an apparently irreversible profile but without any evidence as to the location of this potentially new allosteric site [14].

**Figure 3:**
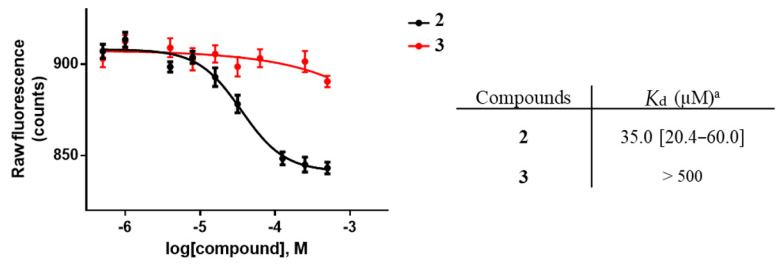
Measurement of binding affinity between **2**, **3** and PHGDH by MST. Dose-response curves of **2** and **3** on isolated PHGDH protein. Binding affinities (*K*_d_) of **2** and **3** (*K*_d_ values were determined from two independent experiments performed in triplicates at each compound dilution, in brackets: 95% confidence intervals).

As a starting point of this study, we decided to detail the binding of **2** using biophysical experiments. Microscale thermophoresis (MST) was thus used to appraise the binding of **2** to PHGDH. The experiments confirmed that 2 binds to PHGDH with a *K*_d_ of 35.02 µM, in the same range as the experimentally determined IC_50_ value. In contrast, the *meta*-substituted analogue **3**, used as a negative control in this experiment, showed no appreciable binding (Figure 3).

Having characterized the interaction of this inhibitor with the enzyme by MST, we then turned our attention to the identification of the site-of-action. Because our attempt to crystallize PHGDH with **2** proved, in our hands, to be unsuccessful, a photo-reactive crosslinking strategy was considered.

### 2.2. Design and Synthesis of the Photoactivatable Probe ***11***

**Scheme 1:**
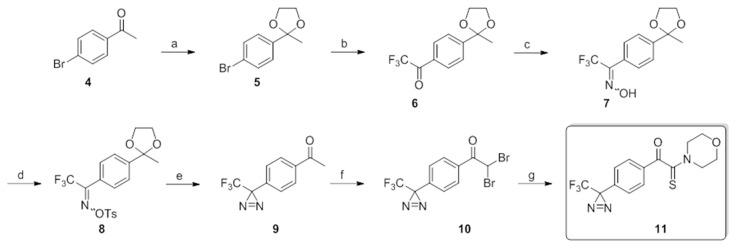
Synthetic pathway of diazirine 11. (**a**) ethylene glycol and PTSA.3H_2_O, toluene, 110 °C, 24 h, quant. (**b**) (i) *n*-BuLi, THF, −78 °C, 2 h (ii) ethyl trifluoroacetate, THF, −78 °C, 3 h, 31%. (**c**) NH_2_OH.HCl, NaOH, EtOH, 78 °C, 18 h, 96%. (**d**) *p*TsCl, Et_3_N, DMAP, CH_2_Cl_2_, rt, 18 h, quant. (**e**) (i) NH_3_/MeOH, THF, rt, 18 h. (ii) I_2_, Et_3_N, MeOH, rt, 1 h. (iii) PTSA, H2O/acetone, rt, 18 h, 50%. (**f**) Br_2_, TBAB, CHCl_3_, rt, 18 h, 89%. (**g**) S_8_, morpholine, DMF, rt, 48 h, 82%.

The reactive photoactivatable probe **11** was designed based on our initial SAR study indicating that the *para*-position on the aryl moiety is preferred. Therefore, we envisaged to incorporate the well-known photoactivatable trifluoromethyl diazirine moiety in this particular *para*-position using the synthetic pathway depicted on Scheme 1 [19].

The chemical synthesis of the target molecule **11** started with the protection of the commercially available ketone **4** to its dioxolane **5** (Scheme 1). Then, a two-step procedure consisting of a lithium/halogen exchange followed by addition of ethyl trifluoroacetate afforded the trifluoromethyl ketone **6**. Condensation of **6** with hydroxylamine and subsequent tosylation led to the tosyl-hydrazone intermediate **8**. The diazirine **9** was further obtained by reacting **8** with ammonia followed by oxidation and deprotection of the ketone. Finally, the bis-α-bromination of **9** led to the dibromoketone **10** that was treated under the Willgerodt–Kindler conditions to deliver the photoactivable probe **11** in good yields.

### 2.3. Evaluation of the Photoactivable Probe ***11***

**Figure 4:**
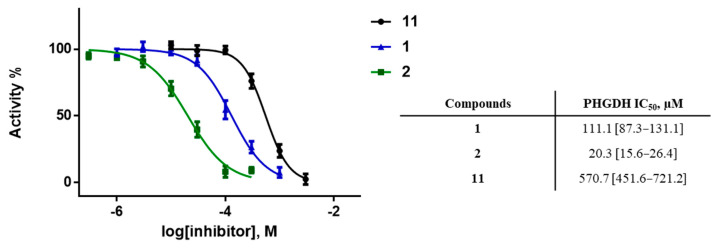
Dose–response curves of compounds **1**, **2**, and **11** on isolated PHGDH. IC_50_ values were determined from two independent experiments performed in triplicates at each compound dilution, in brackets: 95% confidence interval.

Before investigating a possible adduct between the photoactivatable probe **11** and PHGDH, its PHGDH inhibition potency was evaluated, and an IC_50_ value of 571 µM was obtained (Figure 4). This lower PHGDH inhibitory potency compared to the parent α-ketothioamide **2** is relatively consistent with the previously identified SARs highlighting the limited possible pharmacomodulations in this position. Still, we hypothesized that although less potent than 2, this tool compound could help us to reveal the binding mode in this series.

### 2.4. Epitope Mapping Analysis

The binding of the photoactivatable diazirine probe **11** with PHGDH was confirmed by epitope mapping carried out by NMR saturation transfer difference (STD) experiments.

**Figure 5:**
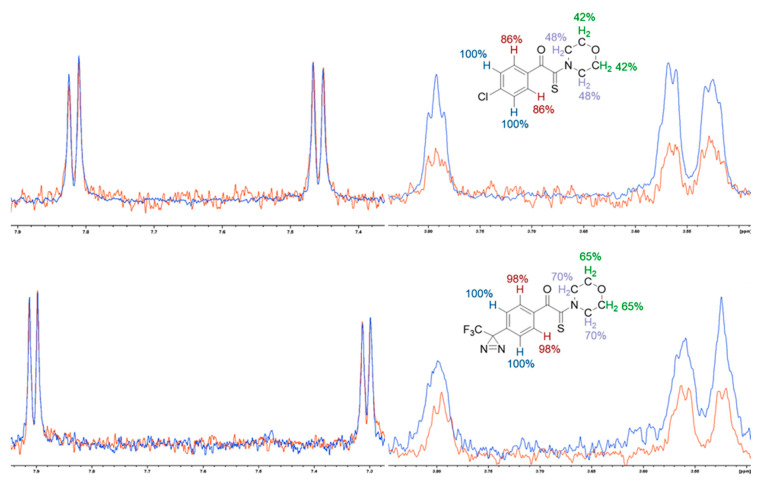
Epitope mapping of **11** and **2**. Superposition of ^1^H (blue) and STD (orange) spectra of **2** (top, 100 µM) and **11** (bottom, 100 µM) with PHGDH (10 µM). The percentages correspond to the relative integration of the STD spectra compared to the ones of the ^1^H spectra. All spectra are scaled in regard to the aromatic protons. A reduction of the signal intensity in the STD spectrum compared to the ^1^H spectrum indicates a lower saturation transfer and hence a weaker interaction.

As depicted in Figure 5, both the photoactivatable diazirine probe **11** and the *para*-chloro substituted compound **2** underwent saturation transfer from the protein via spin diffusion through the nuclear Overhauser effect, corroborating their binding to PHGDH. Furthermore, epitope mapping of both molecules demonstrated a similar pattern of interaction. Epitope mapping corresponds to the relative saturation transfer intensity between the different protons of the molecule. Briefly, the ligands hydrogens in close contact with the protein will undergo a higher saturation transfer than the protons less involved in the interaction and hence display a more intense STD spectrum. Both molecules thus displayed similar binding epitopes, with a high relative saturation transfer for the aromatic hydrogens (Figure 5, blue and red) and a lower relative saturation transfer for the morpholine hydrogens (Figure 5, light blue and green) suggesting a similar interaction pattern for **2** and **11** with PHGDH. This interesting pattern also explains, at least in part, why modification/substitution of this aromatic ring has a considerable impact on PHGDH inhibition, as previously reported [18].

### 2.5. Photoactivation and Mass Spectrometry Analysis

Photoactivation experiments were performed with the diazirine **11** in the presence or absence of **2** at various concentrations to analyze the potential competition for the same binding site of these two compounds.

**Figure 6:**
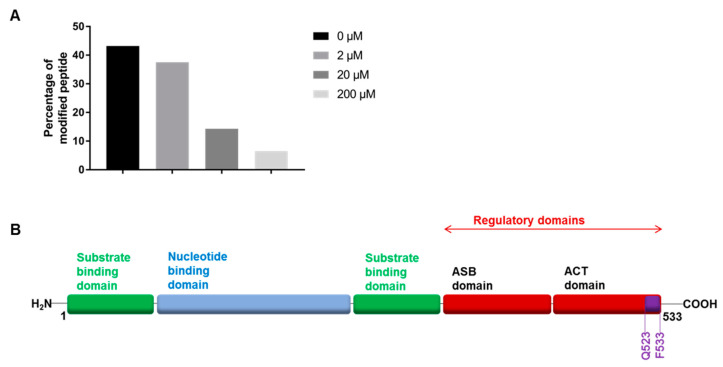
Result of the photoactivation experiment. (**A**) Percentage of the modified QHVTEAFQFHF peptide after incubation with compound **2** at different concentrations determined from the peptide spectrum matches (PSMs) of their precursors after trypsinization and analysis by nanoUHPLC/MS. Compound **2** was incubated with PHGDH in 50 mM Tris and 1 mM EDTA at pH 8.5 during 1 h before adding 1 mM of 11. (**B**) Schematic structure of full-length human PHGDH with substrate-binding domain in green, nucleotide-binding domain in blue, regulatory domains in red and diazirine modified peptide in purple. Adapted from Ravez et al. [10].

Briefly, PHGDH was incubated with or without **2** for one hour, and then the diazirine derivative **11** (1 mM) was added for 30 min. The labeling with the photoactivatable diazirine was performed by irradiation at 350 nm for 10 min. Finally, the enzyme-inhibitor complex underwent trypsin digestion, and the resulting peptides were analyzed by nanoUPLC/MS. As a result, several peptides were identified, some of them being covalently modified by the diazirine **11** (see Table S1 in Supporting Information). Interestingly, among the detected peptides, only the ^523^QHVTEAFQFHF^533^ C-terminal peptide, that is part of the ACT (aspartate kinase-chorismate mutase-TyrA) domain [20] of PHGDH (Figure 6A), could be titrated out by addition with the competing compound **2**. Indeed, only the percentage of labeling of this particular peptide was reduced (from 43% to 6%) upon addition of **2**, thus suggesting that this particular peptide sequence is most probably part of an allosteric binding site for the α-ketothioamide **2** (Table S2 in Supporting Information).

To the best of our knowledge, this ACT domain in PHGDH has never been reported before as a potential target binding site for PHGDH. Interestingly, in lower organisms like *E. Coli*, this domain is a regulatory domain where L-serine binds to induce a negative feedback inhibition of the enzyme (Figure 6B). However, in human PHGDH (Type I enzyme), this serine inhibition no longer occurs due to mutations of key binding residues [20]. The allosteric inhibition by our α-ketothioamides, even if they act on the ACT domain, should therefore be linked to a different and novel mechanism.

### 2.6. Protein Truncation Experiments

**Figure 7:**
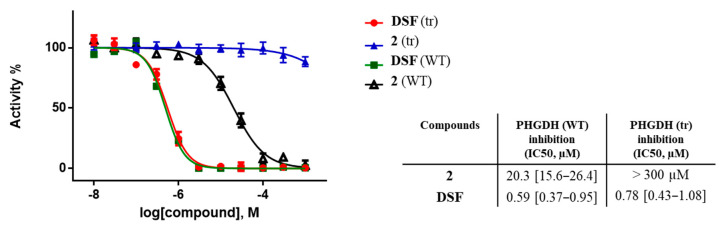
Dose–response curves of compounds **2** and DSF on isolated PHGDH (WT and tr). IC_50_ values were determined from two independent experiments performed in triplicates at each compound dilution, in brackets: 95% confidence interval.

Finally, to further document our hypothesis that **2** binds to the allosteric site on PHGDH delineated by the ^523^QHVTEAFQFHF^533^ C-terminal peptide, that is part of the ACT domain, truncation experiments were conducted. To this end, a PHGDH truncated form lacking the ^523^QHVTEAFQFHF^533^ C-terminal site (PHGDH-tr) was produced. Interestingly, this mutant retained its enzyme activity in the same range to that of the native PHGDH. However, the inhibitory potency of **2** on the mutant was found to be completely abolished (Figure 7), hence demonstrating that the ^523^QHVTEAFQFHF^533^ C-terminal site is indeed most probably the target of **2**. As a comparison, disulfiram (DSF), a well-known PHGDH inhibitor acting at an allosteric site distant from the site identified herein [15], displayed the same PHGDH inhibitory potency both for the native and truncated enzymes.

## 3. Materials and Methods

General Chemistry—All reagents were purchased from chemical suppliers and used without purification. The characterizations of the products were performed as reported previously [14].

PHGDH Assay—Enzymatic assay was carried out following a previously described procedure [18].

PHGDH-tr Purification—pET28a human PHGDH-tr (Genecust) were transformed into BL21 Escherichia coli, the protein was produced and tested following a reported procedure [15]. Protein purity was assessed via SDS/PAGE and Coomassie staining.

MST assay—MST measurements were adapted from a previously reported procedure [21]. Measurements were performed in 50 mM Tris buffer pH 8.5, 250 mM NaCl, 10 mM, MgCl2, and 100 µM DTT containing 0.05% P-20 in premium-treated capillaries (NanoTemper Technologies, Munich, Germany). Experiments were performed in triplicates using 40% LED power, medium MST power, LaserOn time was 20 s, Laser Off time 3 s.

Diazirine irradiation—Human PHGDH (5 µg) was incubated 30 min at room temperature with compound **11** and compound **2** at various concentrations in classical assay buffer (50 mM Tris, pH 8.5, and 1 mM EDTA). The mixture was then irradiated on ice using Stratalinker UV Crosslinker 1800 at 350 nm for 10 min.

Mass spectrometry experiments, Orbitrap Lumos—Mass spectrometry experiments were carried out on an Orbitrap Lumos, following a previously reported procedure, with some modifications [15]. Oxidation on Met; carbamidomethyl (+57.021 Da) on Cys and diazirine (+315.068) on Cys, His, Phe, and Met were considered as variable modifications. All modified peptide sequences were manually validated.

Epitope mapping by Nuclear Magnetic Resonance—All experiments were adapted from a previously published procedure [21]. For 1D Saturation transfer difference (STD) studies, samples were prepared in PBS with 5% d6-DMSO. The concentration of PHGDH was 10 μM. Ligand binding was detected using a STD stddiffesgp.3 sequence with a 2 s saturation time. For each experiment, 256 scans were collected both for on and off resonance experiments (respectively at 0 and 40 ppm).

## 4. Conclusions

In this study, we aimed to detail the site of action of a series of α-ketothioamides on PHGDH to fuel the development of new and potent anticancer drugs acting along a potentially completely novel mechanism-of-action. First, we characterized the binding of our lead compound 2 using microscale thermophoresis experiments. Then, a crosslinking strategy using an original photoactivatable diazirine probe was used to decipher the binding of **2** on PHGDH. Interestingly, this led to the identification of a previously unknown allosteric site on PHGDH delineated by the ^523^QHVTEAFQFHF^533^ C-terminal peptide sequence, that is part of the PHGDH ACT domain, as the target binding site. We further confirmed our hypothesis using a complementary protein truncation experiment.

Although to our knowledge, the structure of the allosteric site identified here is not known, our data pave the way to the development of novel anticancer agents acting along a particularly original mechanism-of-action.

## Data Availability

The data presented in this study are available on request from the corresponding author.

## Supplementary Materials

The following are available online: Chemical synthesis and characterization for **1–11**. Identified peptide residues of PHGDH before and after titration. NMR and HPLC spectrum for **2** and **11**.

## Sample Availability

Samples of the compounds **1**, **2**, **3**, and **11** are available from the authors.
