# Supplementary material for: Unravelling the Allosteric Targeting of PHGDH at the ACT-Binding Domain with a Photoactivatable Diazirine Probe and Mass Spectrometry Experiments
Source: Molecules. 2021 Jan 18;26(2):477. doi: 10.3390/molecules26020477 (PMC7835887; doi:10.3390/molecules26020477)
Supplement: Supplementary file 1 [file molecules-26-00477-s001.pdf]

Supplementary Info File

Unravelling the allosteric targeting of PHGDH at the ACT-binding domain with a photoactivatable dizarine probe and mass spectrometry experiments

Quentin Spillier[1,2], Séverine Ravez[3], Simon Dochain[1], Didier Vertommen[4], Léopold Thabault[1,2], Julien R.C. Prévost[1], Olivier Feron[2], Raphaël Frédérick[1*]

CONTENT

Synthetic procedure of compounds **1-3** S2
Synthetic procedure of diazirine derivative **11** S4
Identified peptide residues of PHGDH (**Table S1**) S6
Peptide residues of PHGDH after titration with **2** (**Table S2**)........ S7
NMR and HPLC spectra data S8
References........ S14

Chemicals

All reagents were purchased from chemical suppliers and used without purification.

Synthetic procedure of compounds 1-3

*Compounds **1-3** were synthesized according the general procedure **I**.*

General procedure I

To a stirred solution of ethanone derivative (1 equiv) in chloroform was added dropwise a solution of dibromine (1.2 equiv) in chloroform. After 2 h, the solvent was evaporated in vacuo to give a crude oil consisting mainly of 2-bromo-1-(substituted)-ethanone compound along with trace amounts of 2,2-dibromo-1-(substituted)-ethanone compound. The mixture was used without purification in the next step. To the synthesized or commercial 2-bromo-1-(substituted)-ethanone derivative were added in sequence DMF, cyclooctasulfur (1.5 equiv), and morpholine (3 equiv). The mixture was then stirred at room temperature. After completion, the reaction mixture was quenched with distilled water to give a precipitate, which was further washed with distilled water. The residue was recrystallized or purified by silica gel chromatography if necessary.

2-Morpholino-1-phenyl-2-thioxoethan-1-one (1). Acetophenone (2.00 g, 16.60 mmol) and dibromine (1.01 mL, 19.90 mmol) were mixed in chloroform (15 mL) to obtain the 2-bromo-1-phenyl-ethanone, and this intermediate was reacted in a second time with morpholine (4.34 mL, 49.80 mmol) and sulfur (0.79 g, 24.90 mmol) in DMF (10 mL). Methanol was used for recrystallization to afford the title compound (2.85 g, 73%). R_f 0.2 (cyclohexane/EtOAc: 8/2). Mp: 110−112 °C. ^{1}H NMR (400 MHz, $CDCl_3$): δH (ppm) 3.59−3.62 (t, 2H, J = 4.8 Hz), 3.69−3.71 (t, 2H, J = 4.8 Hz), 3.90−3.92 (t, 2H, J = 4.8 Hz), 4.33−4.35 (t, 2H, J = 4.8 Hz), 7.48− 7.52 (m, 2 ArH), 7.59−7.65 (m, 1 ArH), 7.99−8.01 (D, 2 ArH, J = 8.2 Hz). ^{13}C NMR (100 MHz, $CDCl_3$): δC (ppm) 47.13, 51.95, 66.40, 66.52, 128.99 (2C), 129.86 (2C), 133.26, 134.48, 187.90 (C=O), 195.70 (C=S). HRMS (ESI$^+$): *m/z* calcd for $C_{12}H_{13}NO_2S$ $(M + H)^+$ 236.0739, found 236.0737. Data are in agreement with the literature.[1]

1-(4-Chlorophenyl)-2-morpholino-2-thioxoethan-1-one (2). This compound was synthesized according to the general procedure I using 2-bromo-1-(2-chlorophenyl)ethanone (0.50 g, 2.14 mmol), morpholine (0.56 mL, 6.42 mmol) and sulfur (0.10 g, 3.21 mmol) in DMF (10 mL). Acetonitrile was used for recrystallization to afford the title compound as a yellow solid (0.22 g, 38%). R_f 0.2 (cyclohexane/EtOAc: 8/2). Mp: 135-137°C. ^{1}H NMR (400 MHz, $CDCl_3$): δH (ppm) 3.58-3.71 (m, 4H), 3.89-3.92 (t, 2H, J = 4.8 Hz), 4.31-4.33 (t, 2H, J = 4.8 Hz), 7.46-7.48 (d, 2 ArH, J = 8.8 Hz), 7.93-7.95 (d, 2 ArH, J = 8.6 Hz). ^{13}C NMR (100 MHz, $CDCl_3$): δC (ppm) 47.18, 51.97, 66.39, 66.53, 129.36 (2C), 131.22 (2C), 131.74, 141.06, 186.45 (C=O), 194.94 (C=S). HRMS (ESI$^+$): *m/z* calcd for $C_{12}H_{13}ClNO_2S$ $(M + H)^+$ 270.0350, found 270.0350. Data are in agreement with the literature.[1]

1-(3-Chlorophenyl)-2-morpholino-2-thioxoethan-1-one (3). This compound was synthesized according to the general procedure I. 1-(3-Chlorophenyl)ethanone (2.00 g, 12.90 mmol) and dibromine (0.78 mL, 15.50 mmol) were mixed in chloroform (15 mL) to obtain the 2-bromo-1-(3-chlorophenyl)-ethanone and this intermediate was reacted in a second time with morpholine (3.38 mL, 38.80 mmol) and sulfur (0.62 g, 19.40 mmol) in DMF (10 mL). The residue was purified by silica gel chromatography

(cyclohexane/EtOAc: 8/2) and the obtained residue was collected by filtration with diethyl ether to give the title compound as a yellow solid (1.50 g, 43%). R_f 0.2 (cyclohexane/EtOAc: 8/2). Mp: 92-94°C. ^{1}H NMR (400 MHz, $CDCl_3$): δH (ppm) 3.59-3.61 (t, 2H, J = 4.8 Hz), 3.70-3.72 (t, 2H, J = 4.8 Hz), 3.90-3.92 (t, 2H, J = 4.8 Hz), 4.31-4.33 (t, 2H,J = 4.8 Hz), 7.42-7.46 (dd, 1 ArH, J= 7.8 Hz), 7.57-7.59 (ddd, 1 ArH, J= 8.0 and 1.0 Hz), 7.85-7.87 (ddd, 1 ArH, J = 7.8 and 1.4 Hz), 7.96-7.97 (dd, 1 ArH, J = 1.8 Hz).^{13}C NMR (100 MHz, $CDCl_3$): δC (ppm) 47.21, 52.00, 66.39, 66.52, 127.98, 129.61, 130.28, 134.33, 135.05, 135.30, 186.07 (C=O), 194.60 (C=S). HRMS (ESI$^+$): m/z calcd for $C_{12}H_{12}ClNO_2S$ $(M + H)^+$ 270.0277, found 270.0278. Data are in agreement with the literature.[1]

__Synthetic procedure of diazirine derivative 11__

2-(4-Bromophenyl)-2-methyl-1,3-dioxolane (5). To a stirring solution of 1-(4-bromophenyl)ethan-1-one (10.0 g, 50.24 mmol, 1.0 equiv.) **4** and ethylene glycol (8.4 ml, 150.72 mmol, 3.0 equiv.) in toluene (140 ml) at room temperature was added PTSA.H_2O (950 mg, 5.0 mmol, 0.1 equiv.). The reaction mixture was stirred at 110°C for 24 h over a Dean-Stark apparatus, allowed to cool down at room temperature, washed with brine (2 x 100 ml), dried over $MgSO_4$, filtered and concentrated on a rotavapor to yield **5** (50.24 mmol, 12.2 g, quant.) as a white crystal and was directly used in the next step. ^1H NMR (400 MHz, $CDCl_3$): δH (ppm) 1.63 (s, 3H), 3.75 (td, 2H, J = 6.1, 4.2 Hz), 4.03 (td, 2H, J = 6.2, 4.2 Hz), 7.30-7.42 (m, 2H), 7.42-7.52 (m, 2H).

2,2,2-Trifluoro-1-(4-(2-methyl-1,3-dioxolan-2-yl)phenyl)ethan-1-one (6). To a stirring solution of bromide **5** (8.13 g, 33.45 mmol, 1.0 equiv.) in THF (330 ml) at -78 °C was added dropwise a solution of n-BuLi (2.26 M in hexane, 31.1 ml, 70.26 mmol, 2.1 equiv.). The reaction mixture was kept at -78 °C for 2 hours and ethyl trifluoroacetate (8.36 ml, 70.26 mmol, 2.1 equiv) in THF (15 ml) was added dropwise to the reaction flask. The temperature was kept between -60°C and -78°C for 3 hours and the reaction quenched with ethanol (10 ml) and water (10 ml) before the temperature was allowed to reach room temperature. Diethyl ether (300 ml) was added and the organic phase was washed twice with brine (2 x 200 ml), dried over Na_2SO_4, filtered and concentrated under reduced pressure. The crude was purified with flash column chromatography (petroleum ether/Et_2O: 6/1) to yield the title compound **6** as a colourless oil (10.37 mmol, 2.74 g, 31%). ^1H NMR (400 MHz, $CDCl_3$): δH (ppm) 1.66 (s, 3H), 3.77 (td, 2H, J = 6.2, 4.2 Hz), 4.08 (td, 2H, J = 6.2, 4.2 Hz), 7.59-7.73 (m, 2H), 8.06 (d, 2H, J = 7.8 Hz).

2,2,2-Trifluoro-1-(4-(2-methyl-1,3-dioxolan-2-yl)phenyl)ethan-1-one oxime (7). Hydroxylamine hydrochloride (1.95 g, 28.04 mmol, 3 equiv.) in ethanol (100 ml) at room temperature was neutralized with an equimolar amount of sodium ethanolate in ethanol (21% w/w, 10.48 ml, 28.04 mmol, 3.0 equiv.). Product **6** was then added to the reaction mixture and the temperature was set to 78°C for 18h, cooled to room temperature, Et_2O was added. The mixture was washed successively with 0.01 M HCl (3 x 50 ml) and water (2 x 40 ml), dried over $MgSO_4$, filtered and concentrated under reduced pressure to afford the oxime **7** (2.48 g, 96%) as a colorless oil. ^1H NMR (400 MHz, $CDCl_3$): δH (ppm) 1.67 (d, 3H, J = 3.5 Hz), 3.68-3.85 (m, 2H), 3.96-4.14 (m, 2H), 7.37-7.73 (m, 4H), 8.94 (bs, 0.5H), 9.17 (bs, 0.5H).

2,2,2-Trifluoro-1-(4-(2-methyl-1,3-dioxolan-2-yl)phenyl)ethan-1-one *O*-tosyl oxime (8). To a solution of oxime **7** (2.48 g, 9.0 mmol, 1.0 equiv.) in DCM (50 ml) at room temperature was added successively triethylamine (2.5 ml, 18.0 mmol, 2.0 equiv.), DMAP (1.21 g, 9.9 mmol, 1.1 equiv.) and pTsCl (1.87 g, 9.9 mmol, 1.1 equiv.). The reaction mixture was stirred at this temperature for 18 hrs, quenched with a saturated aqueous solution of NH_4Cl, the phases were separated and the organic phase was washed with H_2O (1 x 30 ml), dried over $MgSO_4$, filtered and concentrated under reduced pressure. The crude mixture was taken in 40 ml of DCM and filtered over silica, concentrated under reduced pressure to afford the desired tosyl-oxime **8** (9.0 mmol, 3.85 g, quant.) as a colorless oil. ^1H NMR (400 MHz, $CDCl_3$): δH (ppm) 1.65 (s, 3H), 2.49 (s, 3H), 3.67-3.96 (m, 2H), 3.97-4.22 (m, 2H), 7.38 (t, 4H, J = 8.3 Hz), 7.59 (d, 2H, J = 8.4 Hz), 7.89 (d, 2H, J = 8.3 Hz).

1-(4-(3-(Trifluoromethyl)-3*H*-diazirin-3-yl)phenyl)ethan-1-one (9). In a solution of tosyl-oxime **8** (3.6 g, 8.0 mmol, 1.0 equiv.) in THF (10 ml) was added ammonia (7 M in MeOH, 11.5 ml, 80.0 mmol, 10.0 equiv.) at room temperature. The reaction mixture was stirred 18 hours at RT, filtered and concentrated under

reduced pressure to give a pale yellow paste. To this paste was added methanol (16 ml), triethylamine (1.66 ml, 12 mmol, 1.5 equiv.) and then small portions of iodine until the purple color remained. The reaction mixture was stirred at RT for 1 hour, Et_2O (25 ml) was added and this organic phase was washed successively with saturated solutions of sodium thiosulfate (20 ml) and ammonium chloride (20 ml), dried over Na_2SO_4, filtered and concentrated under reduced pressure. The crude mixture was diluted in acetone (30 ml) and water (2 ml) and PTSA.H_2O (3g, 16 mmol, 2.0 equiv.) were successively added. The reaction mixture was stirred at RT for 18 hours, diluted with Et_2O (30 ml), washed with a saturated aqueous solution of $NaHCO_3$ (2 x 30 ml), dried over Na_2SO_4, filtered and concentrated under reduced pressure. After purification by flash column chromatography on silica gel (PE/Et_2O 15:1), the diazirine **9** (4.0 mmol, 1.08 g, 50%) was obtained as a yellow oil. ^{1}H NMR (400 MHz, $CDCl_3$): δH (ppm) 2.61 (s, 3H), 7.28 (d, 2H, J = 9.1 Hz), 7.98 (d, 2H, J = 8.6 Hz). ^{13}C NMR (100 MHz, CDCl3): δC (ppm) 26.65, 120.49, 123.22, 126.55, 126.57, 128.60, 133.84, 137.66, 196.96 (C=O).

2,2-Dibromo-1-(4-(3-(trifluoromethyl)-3*H*-diazirin-3-yl)phenyl)ethan-1-one (10). To a solution of the ketone **9** (456 mg, 2.0 mmol, 1.0 equiv.) in chloroform (20 ml) at RT was added TBAB (322mg, 1 mmol, 0.5 equiv.) and Br_2 (0.22 ml, 4.2 mmol, 2.1 equiv.). The reaction mixture was stirred at this temperature for 18 hrs, diluted with CH_2Cl_2 (30 ml) and washed with a saturated aqueous solution of sodium thiosulfate (20 ml) then water (20 ml), dried over $MgSO_4$, filtered and concentrated under reduced pressure. The product was purified over flash column chromatography on silica gel (PE/Et_2O 15:1) to afford the desired product **10** (1.78 mmol, 690 mg, 89%) as a yellow oil. ^{1}H NMR (400 MHz, $CDCl_3$): δH (ppm) 6.59 (s, 1H), 7.31 (d, 2H, J = 8.3 Hz), 8.15 (d, 2H, J = 8.7 Hz).

2-Morpholino-2-thioxo-1-(4-(3-(trifluoromethyl)-3*H*-diazirin-3-yl)phenyl)ethan-1-one (11). To a solution of dibromide **10** (690 mg, 1.79 mmol, 1.0 equiv.) in DMF (18 ml) was added morpholine (0.47 ml, 5.36 mmol, 3.0 equiv.) and sulfur (86 mg, 2.68 mmol, 1.5 equiv.). The reaction mixture was stirred 48 hours at this temperature, quenched with a saturated aqueous solution of sodium thiosulfate (20 ml) and extracted with CH_2Cl_2 (2 x 50 ml). The combined organic layers were washed with water, dried over $MgSO_4$, filtrated and concentrated under reduced pressure. The product was purified over flash column chromatography on silica gel (CH_2Cl_2/cyclohexane: 1/1 then CH_2Cl_2 100%) to afford the desired product **11** (1.47 mmol, 505 mg, 82%) as a yellow solid. R_f 0.5 (Cyclohexane/EtOAc: 3/2). Mp: 121-123°C. ^{1}H NMR (400 MHz, $CDCl_3$): δH (ppm) 3.58-3.60 (m, 2H), 3.69-3.72 (m, 2H), 3.90-3.92 (m, 2H), 4.31-4.34 (m, 2H), 7.29 (d, 2H, J = 8.3 Hz), 8.02 (d, 2 H, J = 8.6 Hz). ^{13}C NMR (100 MHz, $CDCl_3$): δC (ppm) 27.85-29.07 (CF_3), 47.20, 51.97, 66.38, 66.53, 120.38, 123.12, 126.82, 130.08 (2C), 134.15, 135.18, 186.10 (C=O), 194.60 (C=S). HRMS (ESI$^+$): *m/z* calcd for $C_{14}H_{13}F_3N_3O_2S$ $(M + H)^+$ 344.06806, found 344.06691.

Table S1. PHGDH peptides (after trypsination) showing a diazirine modification after treatment at 1 mM of **11**.

Peptide sequence[a]	m/z observed MH+ (Da)	m/z calculated MH+ (Da)	ʌ (ppm)	Chemical modification
^{59}VTADVINAAEK^{69}	1130.60723	1130.60518	1.81	/
^{59}VTADVINAAEK^{69}	1445.66509	1445.67318	-5.6	Diazirine
^{76}AGTGVDNVDLEAATR^{90}	1488.73076	1488.72888	1.27	/
^{76}AGTGVDNVDLEAATR^{90}	1803.79106	1803.79688	-3.22	Diazirine
^{120}QIPQATASMK^{129}	1074.56621	1074.56121	4.66	/
^{120}QIPQATASMK^{129}	1389.62029	1389.62921	-6.42	Diazirine
^{352}GTIQVITQGTSLK^{364}	1345.77104	1345.76856	1.85	/
^{352}GTIQVITQGTSLK^{364}	1660.84599	1660.83656	5.68	Diazirine
^{470}TQTSDPAMLPTMIGLL AEAGVR^{491}	2272.06311	2272.16756	3.17	/
^{470}TQTSDPAMLPTMIGLL AEAGVR^{491}	2587.23172	2587.23556	-1.48	Diazirine
^{523}QHVTEAFQFHF^{533}	1390.65752	1390.65386	2.63	/
^{523}QHVTEAFQFHF^{533}	1705.71256	1705.72186	-5.45	Diazirine

[a]Superscripted numbers indicate the amino acid numbering of human PHGDH.

Table S2. PHGDH peptides (after trypsination) showing a diazirine modification after treatment at 1 mM of **11** and **A.** 0 µM **B.** 2 µM and **C.** 20 µM **D.** 200 µM of **2**. Percentage of modified peptides was obtained based on the PSMs ratio between modified and unmodified peptides. [a]Superscripted numbers indicate the amino acid numbering of human PHGDH.

**Peptide sequence[a]**	**m/z observed MH+ (Da)**	**m/z calculated MH+ (Da)**	**Λ (ppm)**	**Percentage of modified peptides**
^{523}QHVTEAFQFHF^{533}	1705.71256	1705.72186	-5.45	43.2 %

A. Compound **2** concentration 0 µM

**Peptide sequence[a]**	**m/z observed MH+ (Da)**	**m/z calculated MH+ (Da)**	**Λ (ppm)**	**Percentage of modified peptides**
^{523}QHVTEAFQFHF^{533}	1705.71292	1705.72186	-5.24	37.5 %

B. Compound **2** concentration 2 µM

**Peptide sequence[a]**	**m/z observed MH+ (Da)**	**m/z calculated MH+ (Da)**	**Λ (ppm)**	**Percentage of modified peptides**
^{523}QHVTEAFQFHF^{533}	1705.71282	1705.72186	-5.30	14.3 %

C. Compound **2** concentration 20 µM

**Peptide sequence[a]**	**m/z observed MH+ (Da)**	**m/z calculated MH+ (Da)**	**Λ (ppm)**	**Percentage of modified peptides**
^{523}QHVTEAFQFHF^{533}	1705.71274	1705.72186	-5.35	6.53 %

D. Compound **2** concentration 200 µM

1-(4-chlorophenyl)-2-morpholino-2-thioxoethan-1-one (**2**)

^{1}H NMR

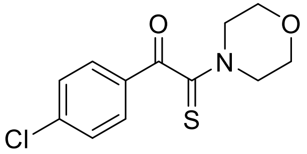

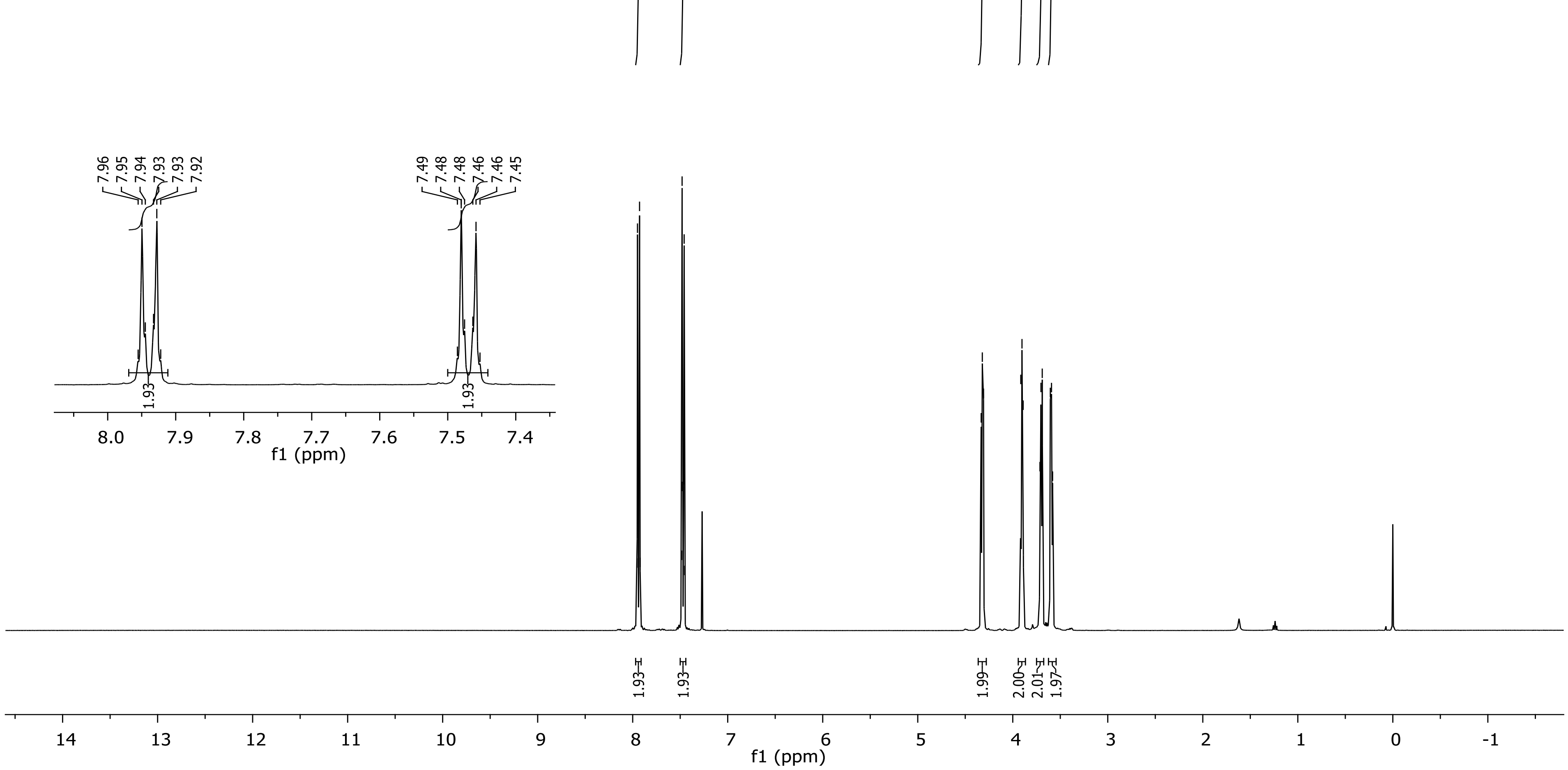

1-(4-chlorophenyl)-2-morpholino-2-thioxoethan-1-one (**2**)

^{13}C NMR

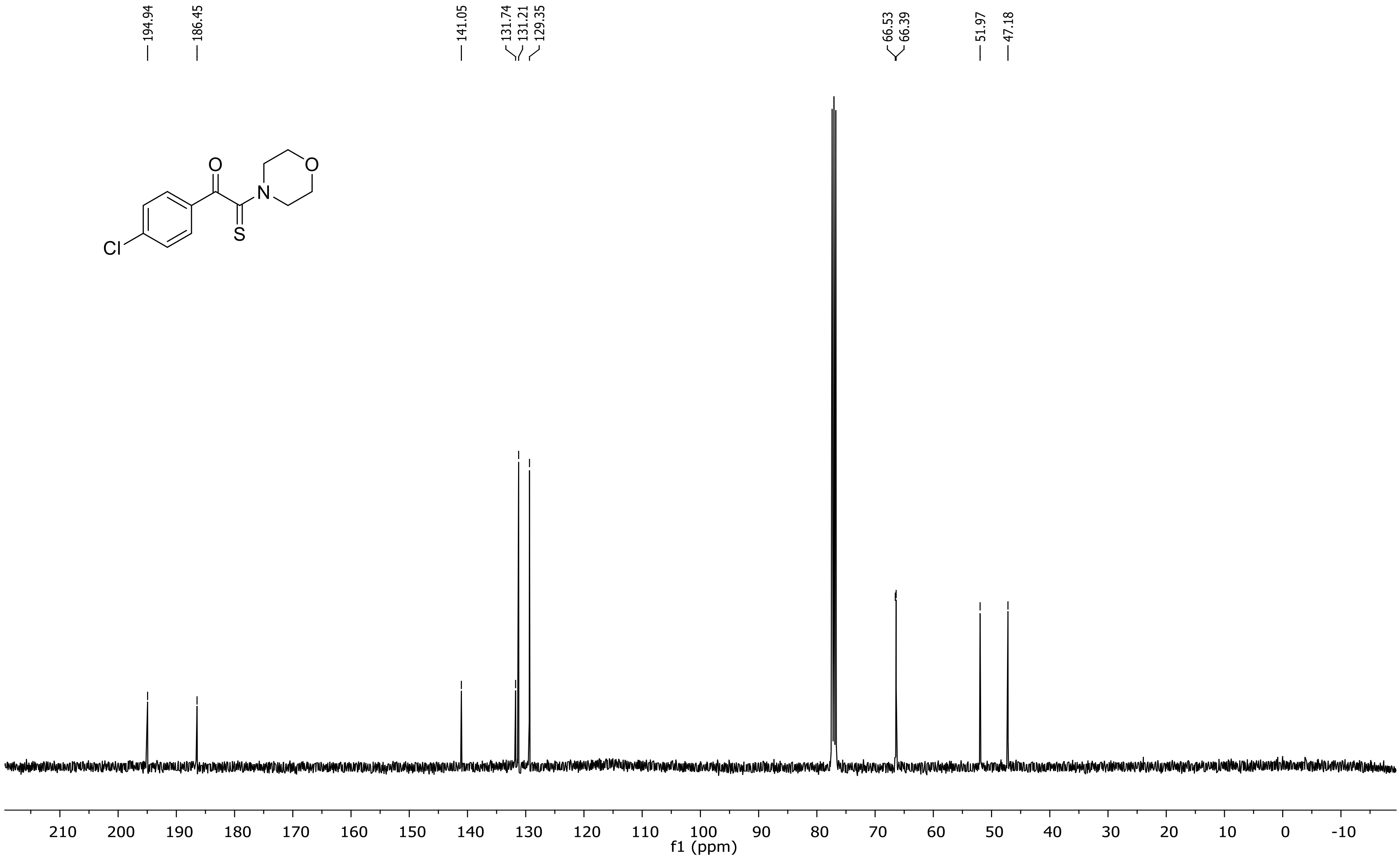

	RT	Area	% Area	Height
1	19.782	7180516	100.00	1142487

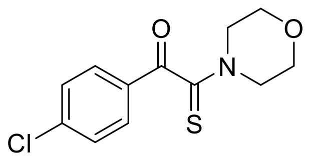

210 nm

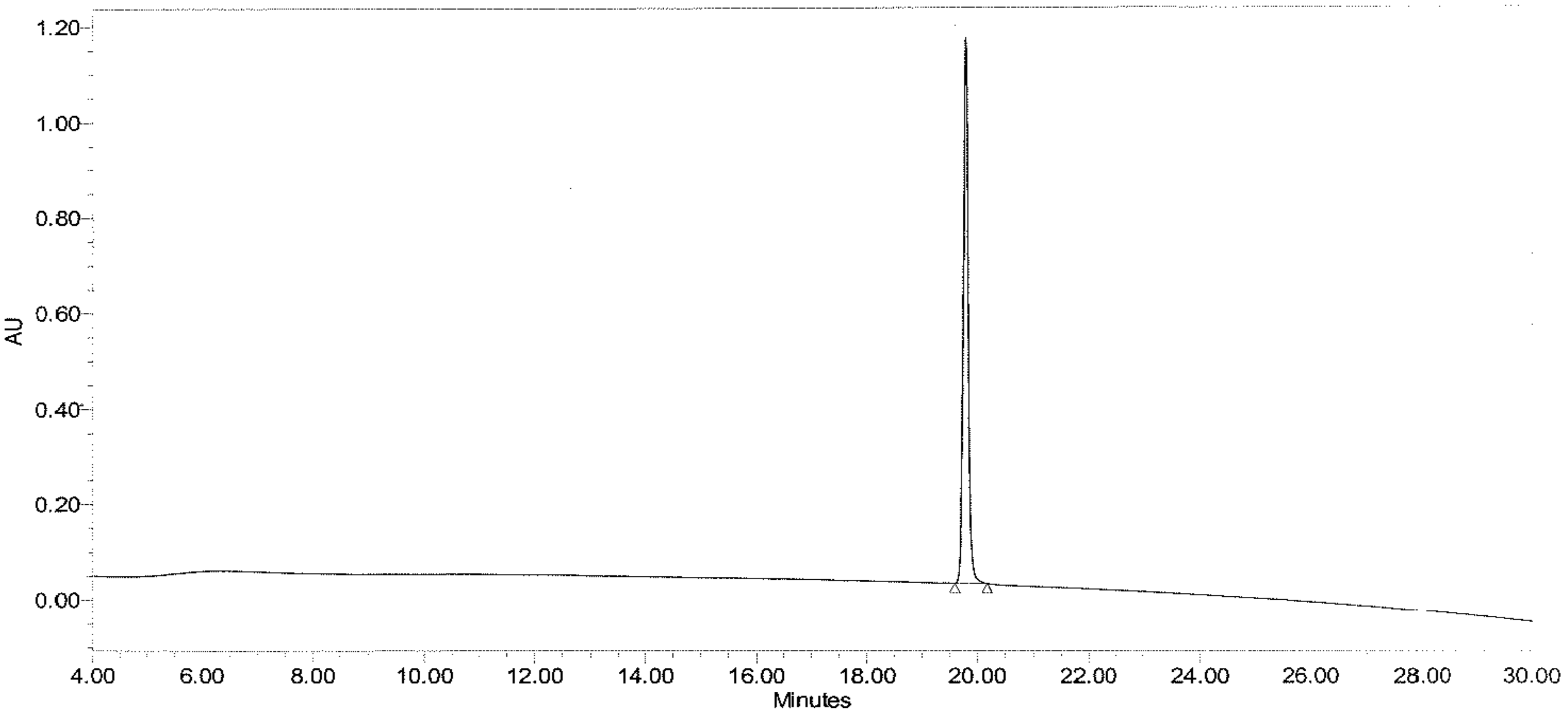

2-morpholino-2-thioxo-1-(4-(3-(trifluoromethyl)-3H-diazirin-3-yl)phenyl)ethan-1-one (**11**)

^{1}H NMR

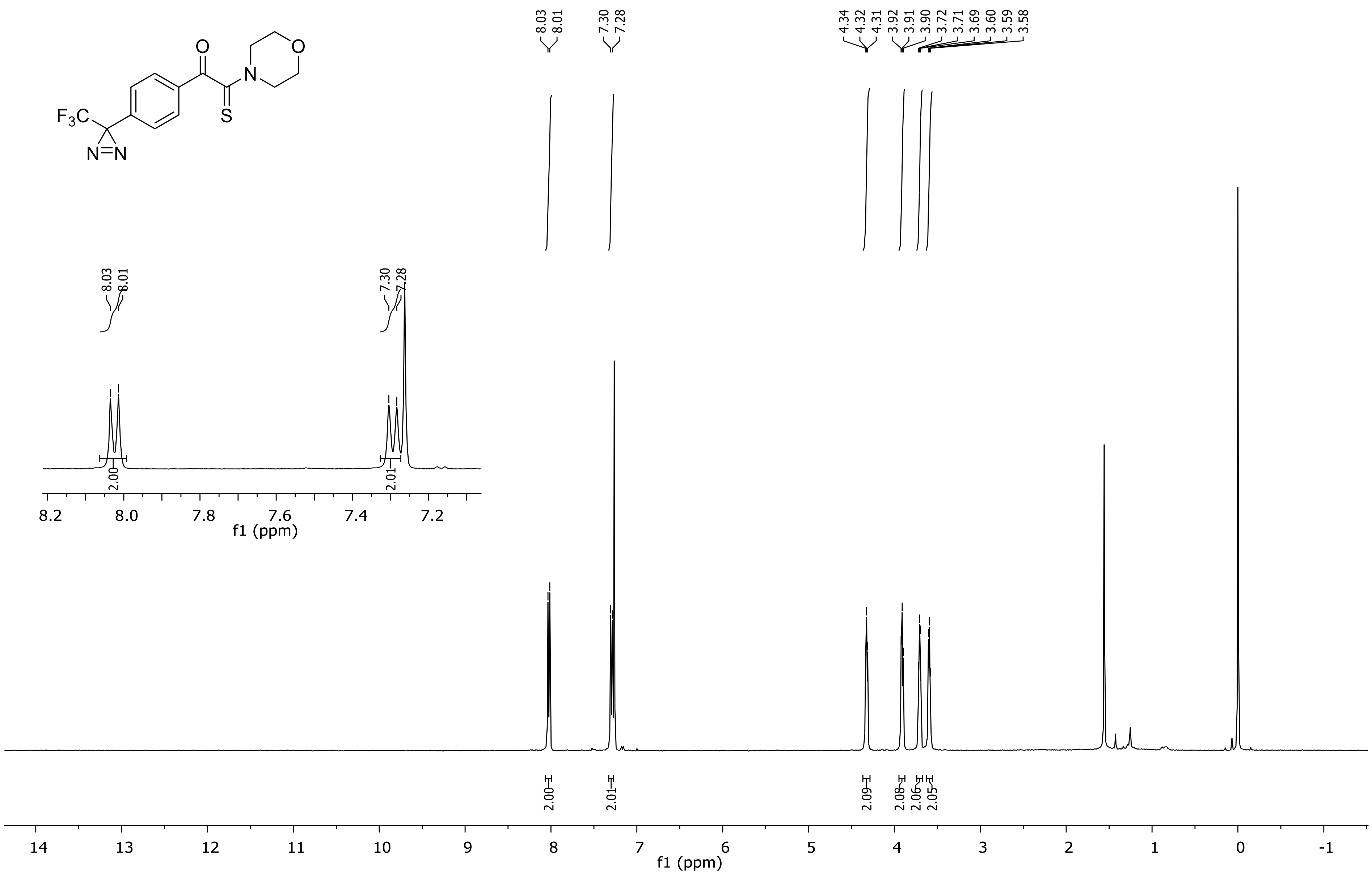

2-morpholino-2-thioxo-1-(4-(3-(trifluoromethyl)-3H-diazirin-3-yl)phenyl)ethan-1-one (**11**)

^{13}C NMR

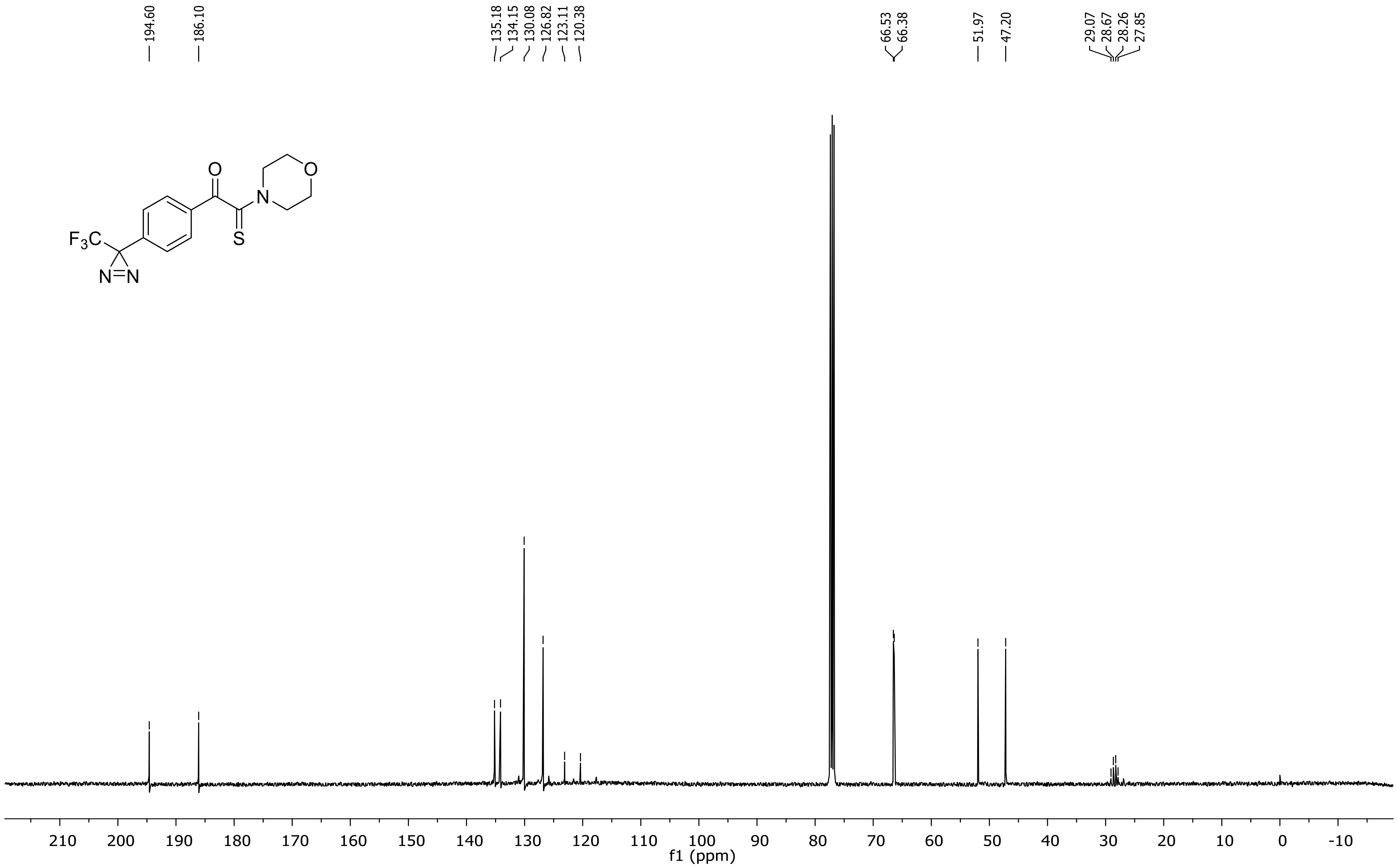

	RT	Area	% Area	Height
1	17.737	59627	1.83	10565
2	22.515	35171	1.08	6466
3	23.640	3155351	97.08	538891

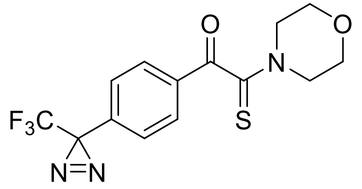

210 nm

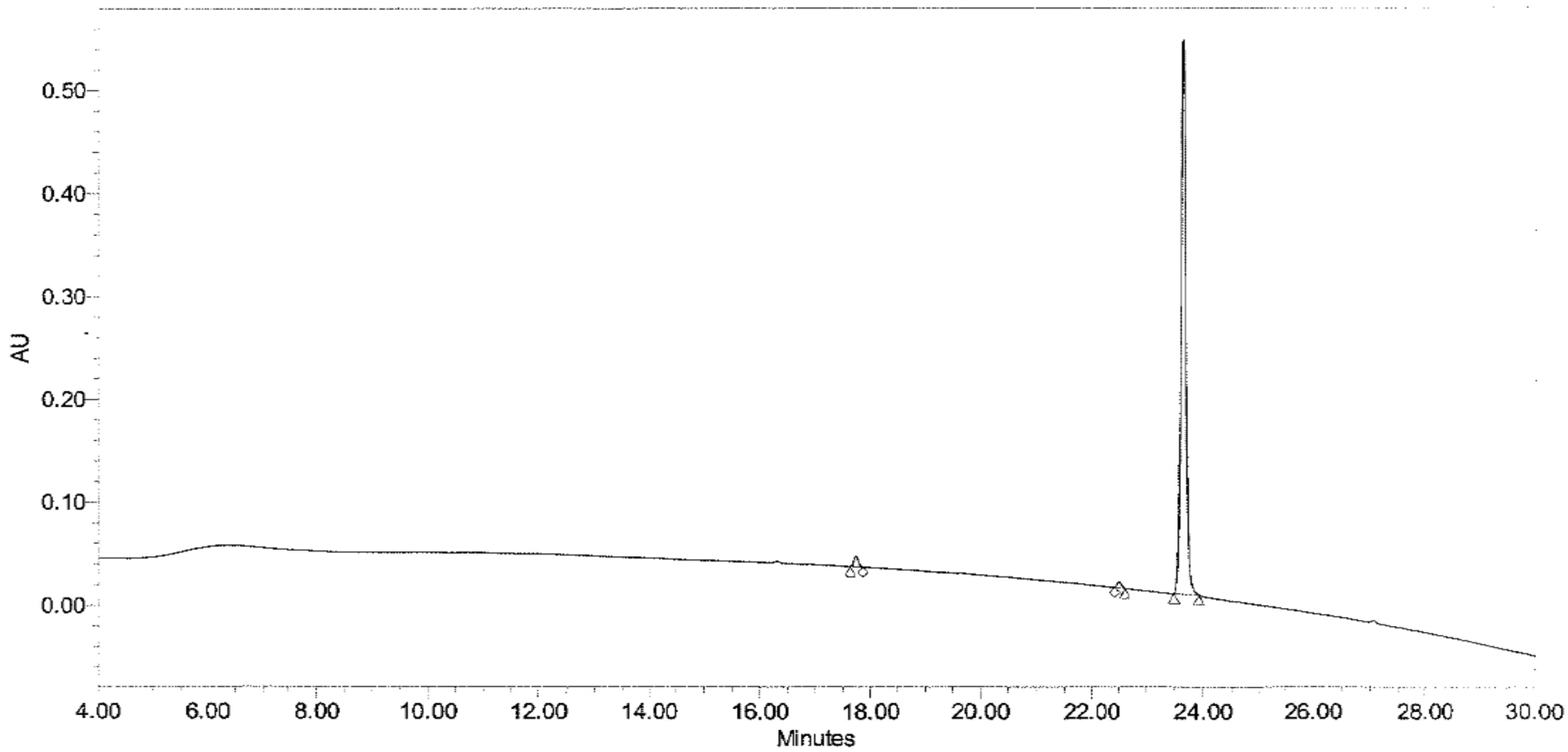

References

1. Ravez, S. *et al.* α-Ketothioamide Derivatives: A Promising Tool to Interrogate Phosphoglycerate Dehydrogenase (PHGDH). *J. Med. Chem.* **60,** 1591–1597 (2017).
